# Effect of an improved agricultural irrigation scheme with a hydraulic structure for crop cultivation in arid northern Afghanistan using the Soil and Water Assessment Tool (SWAT)

**DOI:** 10.1038/s41598-022-09318-2

**Published:** 2022-03-25

**Authors:** Wahidullah Hussainzada, Han Soo Lee

**Affiliations:** 1 Transdisciplinary Science and Engineering Program, Graduate School of Advanced Science and Engineering, Hiroshima University, 1-5-1 Kagamiyama, Higashi-Hiroshima, Hiroshima, 739-8529 Japan; 2 Water and Environment Engineering Department, Mining and Environment Engineering Faculty, Balkh University, Mazar-i-Sharif, Afghanistan

**Keywords:** Hydrology, Civil engineering

## Abstract

The current study focuses on water scarcity, water shortages, and inequal water allocation for downstream water users in the Balkhab River basin (BRB) in northern Afghanistan. The Soil and Water Assessment Tool (SWAT) was utilized to determine the hydrological process in the watershed and assess the water resource capacity. The model was calibrated and validated to ensure proper model setup for the entire watershed. The analysis of the current water management and allocation scheme indicated inadequate water distributions for the downstream irrigation canals. The current water allocation approach was modified based on crop water requirements and the available agricultural lands. A new irrigation scheme was proposed and included in the SWAT model that does not decrease upstream water allocation. The annual streamflow in the Balkhab River can supply the extra allocated water downstream without influencing the upstream water. Notably, a dam was proposed in the middle stream to store water during the winter and early spring seasons, as well as floodwater. The model outcomes showed that the existing annual streamflow in the river can fully support the irrigation of currently available land and an extra 18,470.6 ha of agricultural lands in the BRB. The results of this study can contribute to scientific evidence-based policy and decision-making processes for sustainable agricultural water resource management and flood control in the study region.

## Introduction

Historically, water resource management has been directly linked to economic development and human activities. In the past century, every continent has experienced water scarcity issues, and global water use has increased two times faster than the rate of population increase^1^. Seckler et al.^2^ studied approaches in 118 countries from 1990 to 2025 and concluded that a quarter of the global population and a third of the population in developing countries will experience severe water scarcity issues in the next century. In particular, people in arid and semiarid regions are more vulnerable to water scarcity problems. Recent studies have reported an increase in drought severity in Afghanistan and its neighbouring countries in Central Asia, including Iran^3–6^. Hence, the proper management of water resources is important for reducing vulnerability and improving economic development and the human standard of living.

Afghanistan, as a landlocked country, highly depends on the agricultural sector. A Afghanistan National Statistics and Information Authority report from 2018 noted that the agricultural sector contributed to 18.6% of the country’s total gross domestic product (GDP) in 2018^7^. Regardless of GDP, approximately 80% of the Afghan population lives in rural areas, and their livelihood is directly or indirectly related to agricultural activities^8^. The water sources for irrigation are mainly surface water and groundwater, and 86% of the irrigation water supply comes from surface water^9^. The irrigation water distribution uses an informal traditional system for generation. The operation of the traditional irrigation system is implemented by water users headed by *mirabs*^10^. *Mirab,* a combined Arabic and Persian word, translates as watermaster, and this term is also used in Iran and Central Asia. *Mirabs* are selected by local farmers and paid a specific amount of wheat for their water allocation and canal maintenance services. They typically spend much time walking the canal system, checking or monitoring the water allocation regime and assigning labour forces from the local community for system maintenance. The *mirab* system is an old tradition that has remained for centuries and survived the period of civil war in Afghanistan. The *mirabs* control the main, secondary, and tertiary canals, but they do not have support from or the authority of higher institutions, such as village and provincial councils or the national government, for water allocation to canals from surface waterbodies^11^.

The agricultural sector in Afghanistan has experienced degradation in the past decades of war and conflict due to the collapse of irrigation infrastructure. As reported by Mahmoodi^8^, Afghanistan has $7.9 \times 10^{6} \;{\text{ha}}$ of arable land, and in 1980, $3.3 \times 10^{6} \;{\text{ha}}$ of land was cultivated; however, just $1.8 \times 10^{6} \;{\text{ha}}$ is currently cultivated due to the collapse of portions of the irrigation infrastructure. Currently, uncertainty in water allocation is another issue in Afghanistan irrigation water management. Upstream water users consume more water than those in downstream areas, and downstream farmers receive a less water than upstream farmers, especially during low-flow years^12^. The current irrigation challenges in Afghanistan can be categorized into two major issues: (1) lack of the proper structure and (2) lack of proper management practices for water resources. To overcome the current problems, Afghanistan needs hard and soft approaches. Hard approaches include the rehabilitation of the existing infrastructure and the construction of new irrigation structures with improved efficiency. Soft approaches refer to policy development for water allocation and management among water users considering location differences in the basin.

As reported by the FAO, in 2016, the number of chronically undernourished people in the world was estimated to have increased to 815 million, up from 777 million in 2015^13^. As stated by Dinar et al.^14^, the food security situation in sub-Saharan Africa, southeastern Asia, and western Asia has worsened, especially in locations that have experienced armed conflict or natural disasters. With the rapid growth of the food demand, water scarcity and natural resource issues have become major global policy concerns. Long-term water resource management requires the capacity to evaluate different scenarios of future conditions considering uncertainty and risk.

The Soil and Water Assessment Tool (SWAT) is a useful tool for performing irrigation and chemical yield assessments in watersheds. Scholars have used the SWAT model to investigate different possible scenarios in irrigation water management and agricultural practice worldwide. For instance, SWAT was utilized to predict the impact of alternative agricultural management practices on water quality and quantity in the State of Saxony, central Germany^15^. In the mentioned work the authors performed a SWAT-based parameter sensitivity analysis to improve the modelling scale, and a model was used to analyses the effects of different management practices (e.g., conventional tillage, conservation tillage, and no tillage) on different crop types. Another study utilized the SWAT model and genetic algorithms to optimize the reservoir operation over two reservoirs in Gang River Basin India^16^. In this study, the authors considered multiple scenarios to optimize the dam operation to satisfy the irrigation water consumption in addition to power generation. Panagopoulos et al.^17^ used SWAT to assess the cost effectiveness (CE) of irrigation water management practices in catchments under water stress in Pinios, Greece, based on six best management practices (BMPs). The authors investigated the CEs of deficit irrigation, precision agriculture, wastewater reuse, conveyance efficiency improvements and combinations of these factors to evaluate the management practices in the study area in the context of water scarcity^17^.

Continuous monitoring of drainage water quantity and quality is essential for better understanding hydrological dynamics in intensely irrigated watersheds^18^. However, long-term data collection is expensive and time consuming^19^. On the other hand, generalization of site-specific experimental results to the regional level in a complex watershed with mixed soil and land use is difficult^19^. In this context using watershed modelling tools and remote sensing datasets can be useful for testing management practices at the watershed level. Traditional water practices are still applied for irrigation water management in developing countries and are accepted by local farmers. The local government in those countries prefers not to change the water management to avoid water conflict between users. Considering the population growth and excessive needs of water consumption the traditional water practice need to be replaced by scientific approaches. Hence, the objective of this article is to utilize watershed computer models to test the best management practice (BMPs) on large watersheds with low dataset availability. In undeveloped watersheds, hydrological models can identify the capacities and weaknesses of water resources prior to detailed study and reduce the costs for further investigations and planning. The findings of this study can also contribute to policy and decision-making processes considering the hydrological conditions of basins and other factors.

## Materials

### Study area

**Figure 1:**
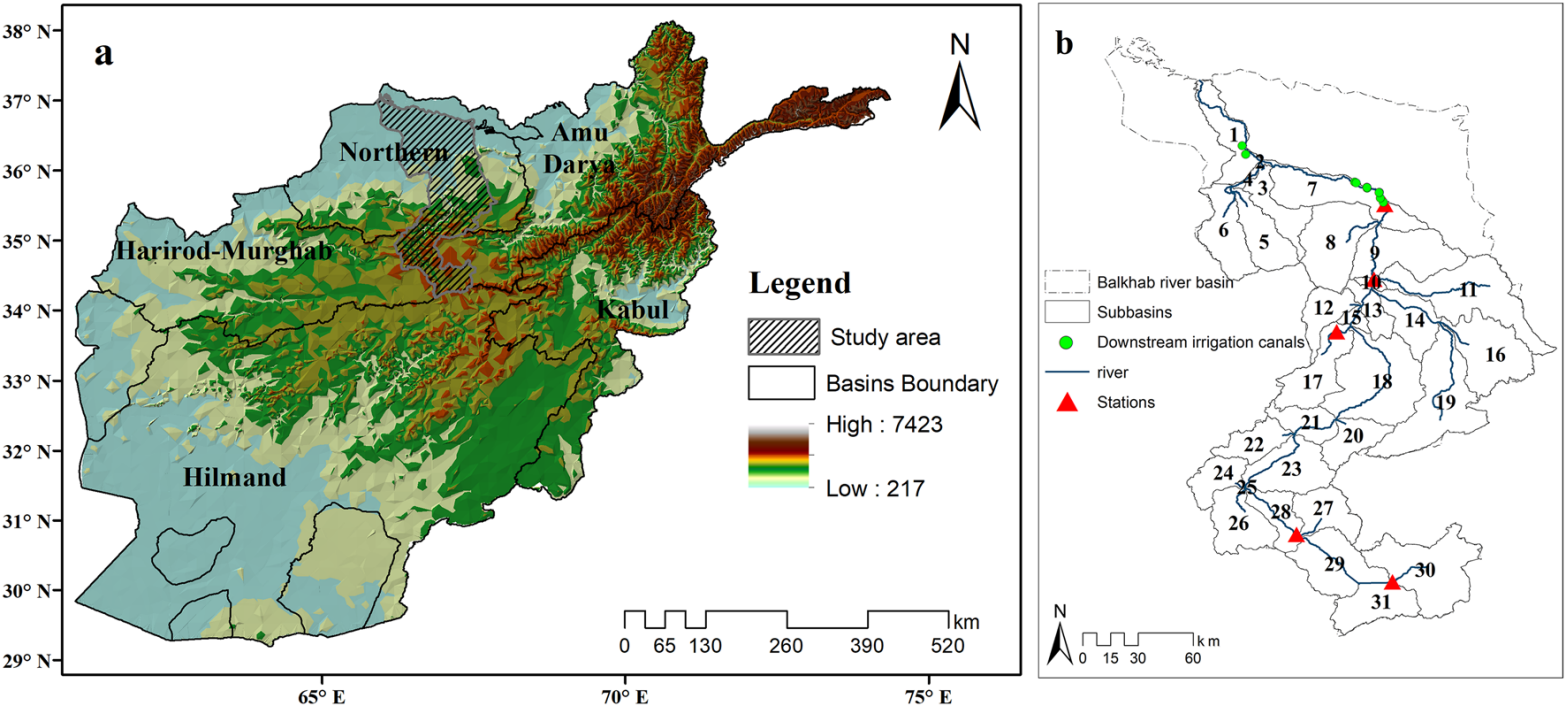
Map of the study area; (**a**) Afghanistan map showing the five major basins and elevation, (**b**) Balkhab River basin (BRB) with the Balkhab River, five hydrometeorological stations, and eleven irrigation canals downstream. (ArcGIS Desktop, Arcmap version10.6 Drawn by author, www.esri.com).

Afghanistan, which has an arid to semiarid climate, is divided into five major basins: the Northern, Harirod Murghab, Helmand, Kabul (Indus), and Amu Darya River basins (Fig. 1a). Furthermore, the NRB is divided into four river basins: Khulm, Balkhab, Sar-i-Pul, and Shirin Tagab. This study was conducted in the BRB (Fig. 1b), which is part of the NRB. The average annual precipitation in the BRB from 2010 to 2018 was 247 mm, and most precipitation occurs in snow form during the winter and as rainfall in the spring^45^. The Balkhab River starts from a series of six lakes in the southern part of the basin and flows through flat lands in the north. Five hydrometeorological stations observe climate data on a daily time scale in the BRB.

The agricultural sector consumes most of the surface water in the BRB to irrigate 296,744 ha of arable land. The irrigation water is distributed through 101 traditional and engineered irrigation canals and 3 small regulator dams located downstream. All available water in the Balkhab River is consumed within the basin or evaporates in the irrigation canals prior to reaching the Afghanistan border. The downstream portion of the BRB is facing a water shortage in the irrigation sector, and the irrigation canals downstream do not receive water or receive inadequate quantities of water for irrigation in six months of the year. Based on the records at the Rabat-i-Bala station on the Balkhab River, the mean annual water volume from 2010 to 2018 was $1657 \times 10^{6} \;{\text{m}}^{3}$, and a considerable amount of flow comes from floods in the early spring season. The water transported during the flood seasons cannot be used since there is no controlling hydraulic structure to store the water for later use.

### Datasets

In this study, SWAT was adopted to develop a hydrological model of the study region and simulate surface runoff. The input datasets used for the model setup were as follows: maximum and minimum temperatures, relative humidity, precipitation, solar radiation, wind speed, a digital elevation model (DEM), land cover, soil type, monthly average discharge, and irrigation canal discharge (Table 1).

**Table 1:** Summary of the datasets used in the SWAT modelling.

Dataset	Temporal resolution	Spatial resolution	Source
Maximum and minimum temperatures, precipitation, relative humidity	Daily	–	Ministry of Energy and Water (MEW), Islamic Republic of Afghanistan
Wind speed and solar radiation	Daily	–	NASA Prediction of Worldwide Energy Resources (https://power.larc.nasa.gov/)
Monthly average discharge	Monthly		Ministry of Energy and Water (MEW), Islamic Republic of Afghanistan
Irrigation canal discharge	–	–	Northern River Basin authority (NRBA)
Digital elevation model	–	12.5 m	ALOS PLASAR (https://search.asf.alaska.edu/#/)
Soil type map	–	–	FAO/UNESCO soil map of the world
Land cover map	–	–	Ministry of Agriculture, Irrigation, and Live stocks (MAIL), Islamic Republic of Afghanistan

**Figure 2:**
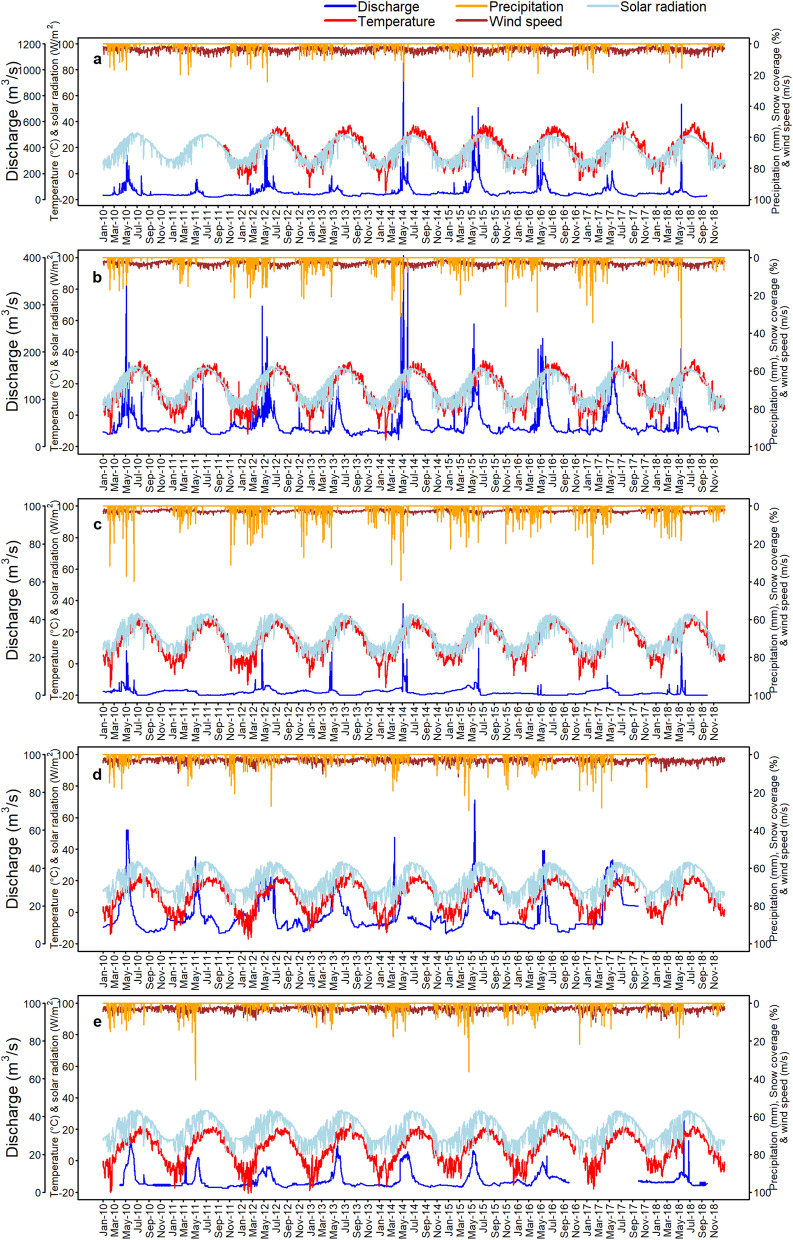
Average daily discharge, temperature, solar radiation, wind speed, precipitation, and snow cover area (%) datasets from 2010 to 2018 at five hydrometeorological stations: (**a**) Rabat-i-Bala, (**b**) Pul-i-Baraq, (**c**) Delmarogh, (**d**) Doshqadam, and (**e**) Nazdik-i-Nayak stations. (R version R- 4.1.2, drawn by author).

Daily climatic data for the maximum and minimum temperatures, relative humidity, precipitation, and river discharge were obtained from the five hydrometeorological stations in the BRB (Fig. 1b). The wind speed and solar radiation datasets were retrieved from the NASA Prediction of World Energy Resources at the same geographical locations as the five stations. The climate datasets from 2010 to 2018 are depicted in Fig. 2.

DEMs with a spatial resolution of 12.5 m were obtained from the ALOS PALASAR Radiometric Terrain Correction (RT1) dataset provided by the Alaska Satellite Facility Distributed Archive Data Center (ASF DAAC). RT1 products are generated from high-resolution and moderate-resolution DEMs^20^. Then, the DEMs were used to create a slope map in the SWAT model.

The land cover map used was retrieved from the Agricultural Market Information Services System of the FAO in collaboration with the Ministry of Agriculture, Irrigation, and Livestock, Islamic Republic of Afghanistan. The land cover map was created using moderate-resolution satellite images from SPOT-4 and the Global Land Survey (GLS) Landsat Thematic Mapper, high-resolution satellite imagery, aerial photographs and ancillary data^21^. Based on the land cover map, the BRB land use is dominated by range land and grassland, with 62.25% coverage; agricultural land covers 23.12% of the basin, and the remaining areas include urban land, barren land, forest, sand/desert, and water bodies.

In many parts of the world, a proper and reliable soil type map does not exist, but the global soil type maps of FAO/UNESCO and the Harmonized World Soil Database (HWSD_v121) can be used instead^22^. The FAO/UNESCO soil map of the world and corresponding data were retrieved and used in this study. This soil map was developed from a statistical analysis of 4353 soil profiles from the World Inventory of Soil Emissions (WISE) database, which was developed at the International Soil References and Information Centre (ISRIC) to provide geographic descriptions of soil factors that influence global change^23^. Further descriptions and information regarding the FAO/UNESCO soil map of the world were provided by Doe^24^.

A large portion of the available water in the Balkhab River is used for agricultural water consumption. The water distributed to the canals is handled by the Northern River Basin Authority (NRBA). The amount of water discharged into the canals was obtained from an official report (NRBA^25^, pp. 24–26). The geographical locations of canal intakes and water rights datasets were obtained for irrigation water management from the report and included in the SWAT model.

### Seasonal crop cultivation

**Figure 3:**
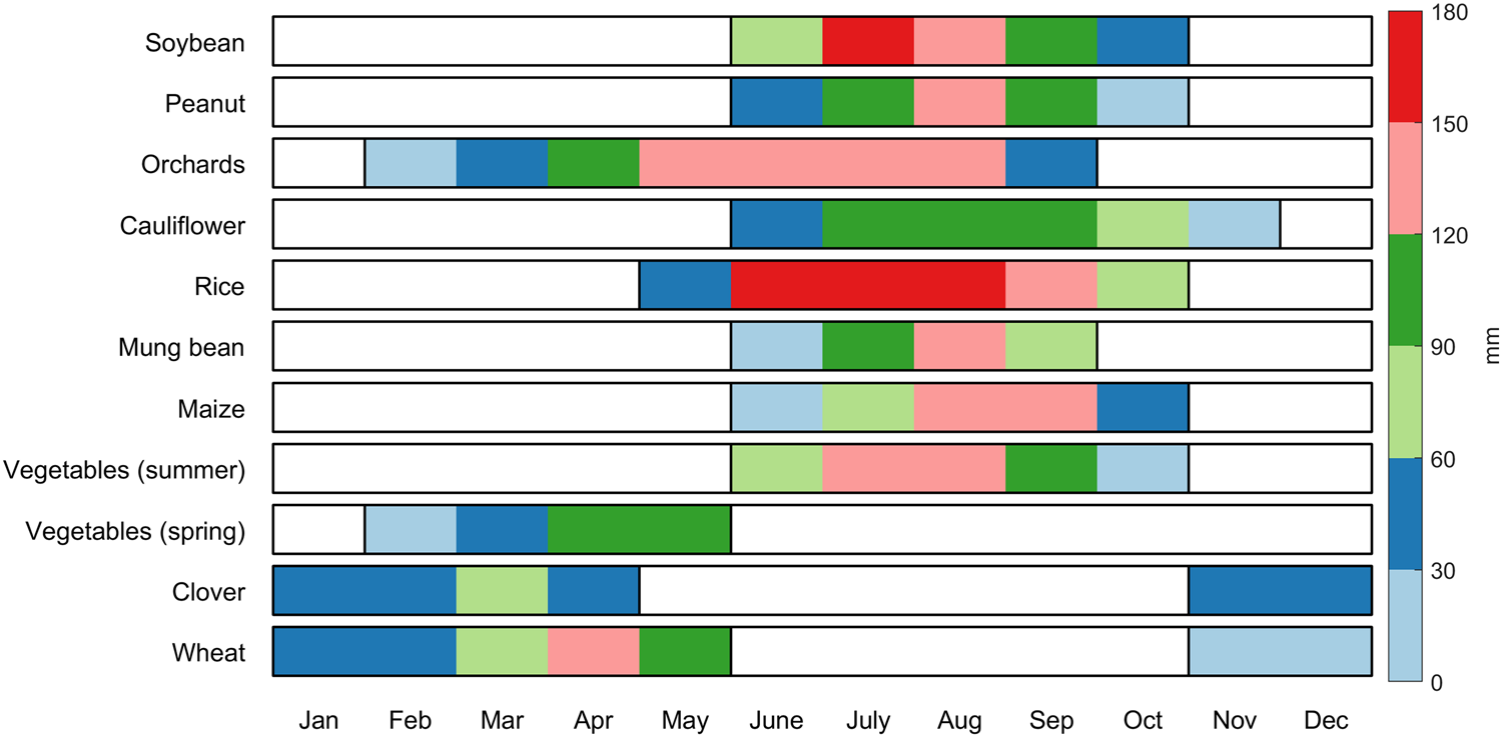
Typical cultivation season by crop type in Khost, Afghanistan, in 2017^28^. The coloured bars indicate the monthly crop water requirements (mm). (R version R- 4.1.2, drawn by author).

The economy in Afghanistan highly depends on the agricultural sector, and 80% of the population lives in rural areas engaged in agricultural activities^8^. As noted by the FAO^26^, the crop types in northern Afghanistan include wheat, barley, rice, maize, pulses, potato, oil seed, clover, lucerne, vegetables, cotton, vegetables, melons, and opium poppy. Proper understanding of crop calendar information is essential for irrigation engineers to achieve synchronization between water delivery patterns and crop life cycles^27^. Such synchronization can contribute to efficient water usage for irrigation. The crop water requirements based on the crop calendar must be considered in irrigation water management and planning. Studies of Afghanistan are limited by data availability in many fields, and irrigation-related data are particularly scarce. In this study, crop water requirements were adopted from a previous study of Khost Province in southern Afghanistan^28^. Wali et al.^28^ used satellite remote sensing data to estimate crop water requirements. The mean annual temperature in Khost Province is 10.4 °C, and the mean annual precipitation is 188 mm; these values are similar to those in the BRB. Figure 3 illustrates the crop calendar based on a field survey and an interview of farmers in 2017 by Wali et al.^28^. The crop water requirements in different months are summarized in Table S1 in the Supplementary material.

## Methodology

### Current irrigation status

The amount of water allocated from the main river to the main irrigation canals is decided by the governmen. The data for water allocation in the main canals are collected by the government and are used in the current study^25^. However, there is an exception for water allocation to downstream canals due to water scarcity issues during the year. Water allocation amounts for the 11 downstream irrigation canals (Table 2) are based on the water availability in the main river. The available water in the main river is distributed based on tax payments and the agricultural land area in different regions for the 11 canals (G. Sakhi, personal communication, November 2, 2020). The water rights for the 11 canals are summarized in Table 2.

**Table 2:** Irrigation land area and water rights for the 11 downstream irrigation canals^25^.

No.	Canal name	Irrigation land area (ha)	Water rights (%)	Canal length (km)
1	Imam Sahib	7741.9	3.80	41.9
2	Nahar-i-Shahi	14,005.2	10.66	41.7
3	Sia Gird	7702.0	2.85	21.7
4	Balkh	8086.6	1.33	18.3
5	Moshtaq	9207.1	3.98	25.5
6	Chemtal	8003.4	3.12	31.9
7	Abdullah	22,866.3	13.32	30.6
8	Dawlat Abad	38,728.2	14.27	28.3
9	Char Bolak	36,078.3	14.27	20
10	Fayaz Abad	17,180.5	11.42	22.8
11	Aqcha	88,706.0	20.94	43.8
Total		258,305.5	100	326.5

### Irrigation scenario

In this study, a new irrigation scenario for the BRB is defined based on the agricultural land area and crop water requirements. The crop type and cultivated land area were determined based on the national agriculture profile from 2016 to 2018. Per the NSIA^7^, the main cultivated crops (and associated cultivation areas) are wheat (60.0%), rice (3.3%), barley (3.3%), maize (3.6%), pulses (2.3%), vegetables (4.3%), potato (1%), onion (0.3%), fruits (9.3%), almond (0.6%), oil seed (2.6%), and others (9.4%). The crop types (and areas) considered in different agricultural land scenarios are wheat (65%), rice (5%), maize (5%), pluses (4%), spring vegetables (5%), summer vegetables (4%), and orchards (12%). In addition, based on the crop water requirements for different crop types (Table S1) and the specified irrigation area for each crop (Table 2), the monthly water requirement for 1 ha of agricultural land is estimated as shown in Table 3.

**Table 3:** Estimated monthly crop water requirement for 1 ha of agricultural land in the downstream BRB area.

Crop type	Area (%)	Monthly crop water requirement (m^3^/ha/mon)											
		January	February	March	April	May	June	July	August	September	October	November	December
Wheat	65	213.9	339	514.2	828.75	610.4						59	111.2
Rice	5					27.85	76.95	85.5	77.4	61.9	31.3		
Maize	5					11	42.4	74.95	60.15	18.8			
Pulses	4					6.44	39.52	59.28	26.72	0			
Summer vegetables	4					35.08	49	53.8	42.04	2.32			
Spring vegetables	5		8.4	25.05	58.2	46.8							
Orchards	12			57	130.8	163.6	166	167.3	147.1	57.5			
Total	100	213.9	348	569.2	1017.8	901.1	373.8	440.8	353.4	140	31.3	59	111.2

A substantial portion of usable water will be lost due to seepage in the irrigation canals^29^. Open canal water losses in Afghanistan irrigation systems are approximately 20%^30,31^. The water losses in irrigation canals placed in natural soil and fine or coarse sediment can range between 20–50%^29^. In this study, the approximate 20% of water losses in the irrigation canals is added to the water allocated for all 11 canals. Considering the data in Tables 2 and 3, as well as the 20% water losses in the irrigation canals the monthly water allocations for the 11 canals were updated based on the new irrigation scenario in the BRB, as shown in Table 4.

**Table 4:** Monthly required water allocations for the 11 downstream irrigation canals based on the monthly crop water requirements and 20% water losses in the irrigation canals.

No.	Canal name	Irrigation land (ha)	Required canal water allocation (m^3^/s)											
			January	February	March	April	May	June	July	August	September	October	November	December
1	Imam Sahib	7741.9	0.74	1.34	1.97	3.65	3.13	1.34	1.53	1.23	0.50	0.11	0.21	0.39
2	Nahar-i-Shahi	14,005.2	1.34	2.42	3.57	6.60	5.65	2.42	2.77	2.22	0.91	0.20	0.38	0.70
3	Sia Gird	7702.0	0.74	1.33	1.96	3.63	3.11	1.33	1.52	1.22	0.50	0.11	0.21	0.38
4	Balkh	8086.6	0.77	1.40	2.06	3.81	3.26	1.40	1.60	1.28	0.52	0.11	0.22	0.40
5	Moshtaq	9207.1	0.88	1.59	2.35	4.34	3.72	1.59	1.82	1.46	0.60	0.13	0.25	0.46
6	Chemtal	8003.4	0.77	1.38	2.04	3.77	3.23	1.39	1.58	1.27	0.52	0.11	0.22	0.40
7	Abdullah	22,866.3	2.19	3.95	5.83	10.77	9.23	3.96	4.52	3.62	1.48	0.32	0.62	1.14
8	Dawlat Abad	38,728.2	3.71	6.69	9.88	18.25	15.64	6.70	7.65	6.13	2.51	0.54	1.06	1.93
9	Char Bolak	36,078.3	3.46	6.23	9.20	17.00	14.57	6.24	7.13	5.71	2.34	0.51	0.99	1.80
10	Fayaz Abad	17,180.5	1.65	2.97	4.38	8.10	6.94	2.97	3.39	2.72	1.11	0.24	0.47	0.86
11	Aqcha	88,706.0	8.50	15.31	22.62	41.80	35.81	15.35	17.52	14.05	5.75	1.24	2.42	4.42
Total		258,305.5	24.75	44.59	65.87	121.71	104.28	44.70	51.01	40.90	16.74	3.62	7.06	12.87

### SWAT model setup with a proposed dam

The SWAT model is a physical process-based semi-distributed hydrological model that was developed to predict the impacts of water management, sediment, and the agricultural chemical yield in large watersheds at the daily scale^32–34^. The model simulates the hydrological process at daily, monthly, or annual time scales based on the daily temperature, precipitation, relative humidity, wind speed and solar radiation. SWAT is a widely accepted tool for efficient water resource assessment^35^ and evaluations of nonpoint source pollution and climate change in relation to water supplies and watersheds^36,37^. The model predicts surface runoff based on a water balance equation (Eq. 1).

1 $$SW_{t} = SW + \mathop \sum \limits_{t = 1}^{t} \left( {R_{i} - Q_{i} - ET_{i} - P_{i} - QR_{i} } \right)$$

where $SW_{t}$ and $SW$ are the respective final and initial soil water contents at time $t$, respectively; $R$, $Q$, $ET$, $P$, and $QR$ are precipitation, runoff, evapotranspiration, percolation, and the return flow, respectively, on day $i$; and all units are mm of H_2_O.

**Figure 4:**
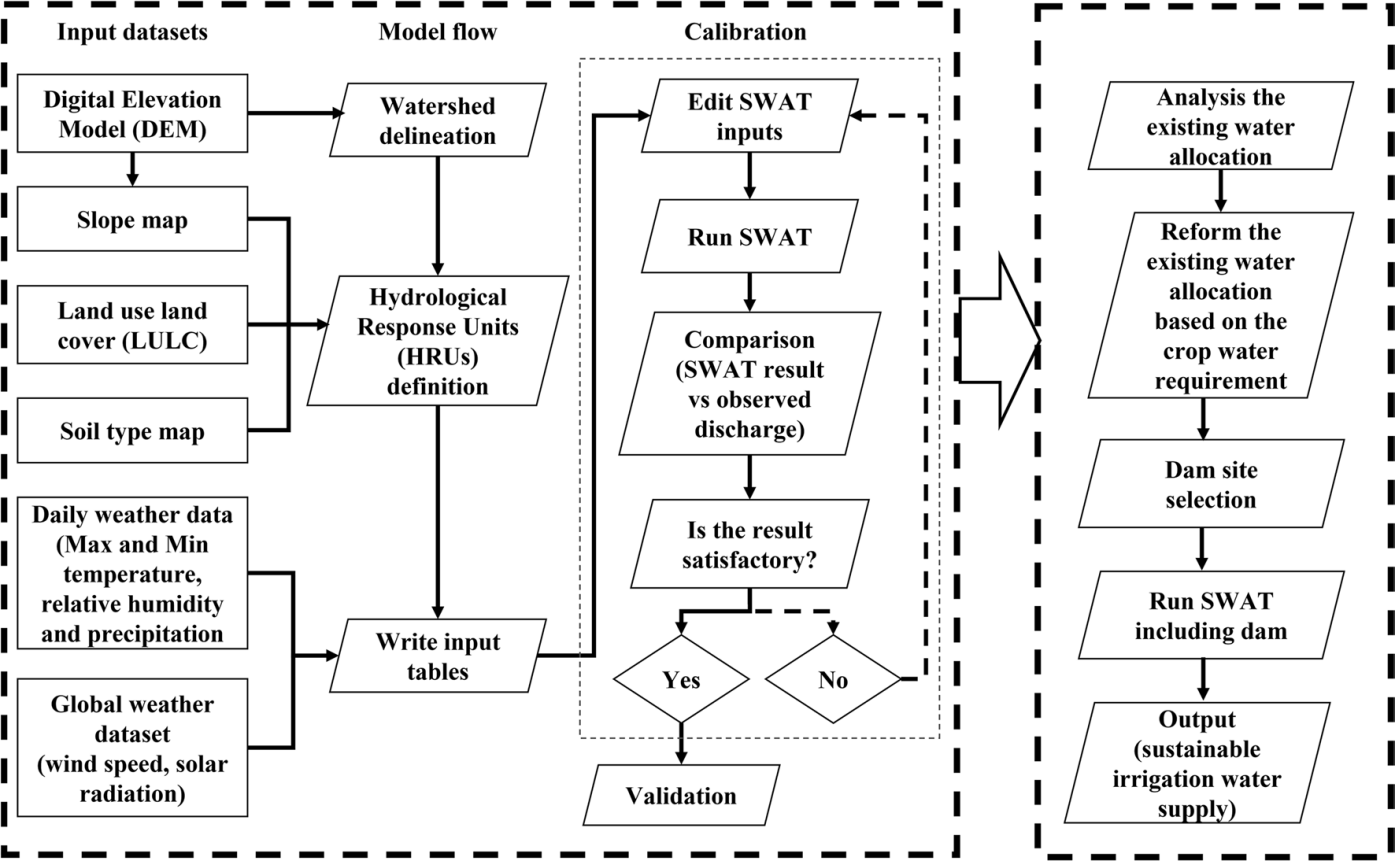
Methodological flow chart for hydrological modelling with SWAT. (PowerPoint, drawn by author).

For reproducibility, the major procedure used for SWAT model setup is summarized in Fig. 4.

*Step 1* Watershed delineation: The SWAT in the ArcGIS interface was used to simulate streamflow and divide the study area into subbasins. In this step, the study area was divided into 31 subbasins, point source data were added to the model, and a dam in subbasin 10 was defined and added. To determine the dam location, three criteria were considered: (1) the slopes of the river and streambed, with a maximum of 5% and minimum of 2% (0.02 < slope < 0.05)^38^, (2) the river discharge, and (3) the horizontal distance from the river within 500 m. Analyses were performed, and subbasin 10 was determined to be the most appropriate site for potential dam construction. However, a more detailed and complex approach is required for final decisions based on socioeconomic criteria in future work. The monthly flow downstream of the dam was estimated based on the monthly required water allocations, as shown in Table 4. The irrigation water consumption from September to December is relatively low to meet the requirements of the downstream environment and water supply; thus, we assume the minimum discharge downstream of the dam during this period at $20\;{\text{m}}^{3} {\text{/s}}$.

*Step 2* Defining hydrological response units (HRUs): Land cover, soil type, and slope maps of the study area were used for further division of 31 subbasins into 1532 smaller units.

*Step 3* Creating input tables: The meteorological data from the five stations from 2010 to 2018 were defined and uploaded to the model database to simulate surface flow in this step.

*Step 4* Calibration and validation: Parameter optimization was performed for the SWAT model using the SWAT Calibration and Uncertainty Procedure (SWAT-CUP) v5.2.1^39^ and the sequential uncertainty fitting algorithm (SUFI2) for model calibration and uncertainty analysis. The SUFI2 details are presented by Homan et al.^40^ and Abbaspour^39^. The SWAT model was run for the BRB with a three-year warm-up period from January 2010 to December 2012. Then, the model was calibrated with data from January 2013 to December 2015 based on the monthly discharge recorded at four stations, namely, Rabat-i-Bala, Pul-i-Baraq, Doshqadam, and Nazdik-i-Nayak, located in subbasins 7, 10, 28, and 30, respectively. Next, the model was validated for the period from January 2016 to September 2018 with the remaining data. The calibrated parameters were adopted from a previous study in the BRB, and for more information on the detailed calibration procedure and optimized parameter values, please refer to the study by Hussainzada and Lee^41^.

## Results

### SWAT model calibration and validation

**Figure 5:**
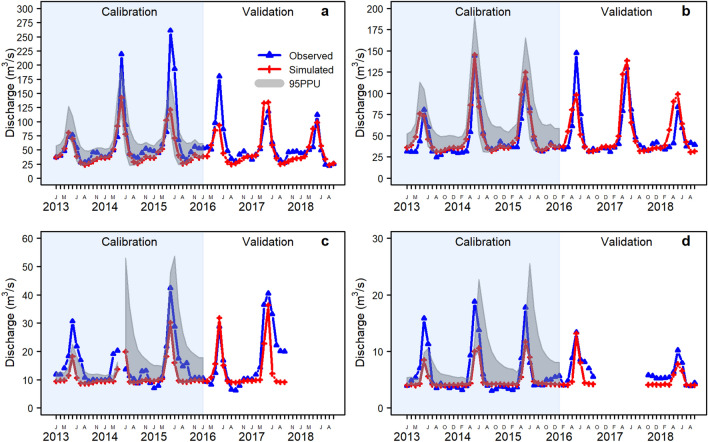
Comparisons between the simulated and observed discharge values in (**a**) subbasin 7, (**b**) subbasin 10, (**c**) subbasin 28, and (**d**) subbasin 30 during calibration and validation periods. (R version R- 4.1.2, drawn by author).

As described, the study period was divided into warmup, calibration, and validation periods, and model calibration was conducted using SWAT-CUP. First, the model was calibrated for the elevation parameters; then, calibration was performed for snow parameters and the seven most sensitive parameters. To address the downstream flow during the dry season from July to September, the groundwater baseflow was estimated based on point-source station-recorded discharge at 190 springs in the BRB. With the estimated baseflow, the SWAT results were markedly improved. The hydrographs in Fig. 5 show the comparison between the simulated and observed monthly discharge values at the four stations in subbasins 7, 10, 28, and 30 (Fig. 1b). The model performance was evaluated using the coefficient of determination (R^2^), Nash–Sutcliffe efficiency (NSE)^42,45^, and percent BIAS (PBAIS)^43^ by comparing the simulated and observed values at the four stations along the river. The simulation results exhibit good agreement with the observed monthly discharges at the stations, and the statistics for model performance are summarized in Table 5. As stated by Moriasi et al.^44^ the model’s performance during the calibration period is categorized as satisfactory in subbasin 28, good in subbasin 7, and very good in subbasins 10 and 30 when considering R^2^ values; in terms of NSE, the model performance is not satisfactory in subbasin 28, satisfactory in subbasins 7 and 30, and very good in subbasin 10; and the model performance is satisfactory at all stations except subbasin 10, which shows a very good performance. While the model performance during the validation period is satisfactory in all subbasins except subbasin 30, which is good considering R^2^, the model performance is satisfactory in subbasins 7 and 10 and unsatisfactory in subbasins 28 and 30 considering NSE, and the PBIAS model performed very well in subbasin 10, good in subbasin 7 and satisfactory in subbasins 28 and 30.

**Table 5:** Summary of statistical indicators for model performance.

Subbasin	Calibration			Validation		
	R^2^	NSE	PBIAS	R^2^	NSE	PBIAS
7	0.70	0.52	23.4	0.63	0.58	10.7
10	0.86	0.83	− 8.5	0.66	0.61	− 4.6
28	0.67	0.40	23.4	0.58	0.47	20.2
30	0.80	0.57	17.5	0.76	0.43	20.2

### Irrigation scenario with a proposed dam

As described, the current water allocations for irrigation canals are determined by the government based on taxes, the agricultural land area and the availability of water in the main river. Available water in the river is distributed to the irrigation canals to meet their needs for the entire year. In the current study, a dam is proposed and added to subbasin 10 as a water storage structure to balance the water flows for irrigation purposes throughout the year. The current water allocation amounts for the 11 irrigation canals were estimated based on the water rights for the canals (Table 2) and the average monthly available river discharge. The available average monthly discharge for the river was retrieved from the SWAT model results for subbasin 10, and the results are summarized in Table 6. The Balkhab River experiences the highest flow in May (113 m^3^/s) due to rapid snowmelt and rainfall^41^. The driest month in the basin is September, with an average total monthly flow of 31.97 m^3^/s.

**Table 6:** Current monthly water allocations based on the average simulated discharge (2013–2018) for the 11 irrigation canals in the BRB.

No.	Canal name	Irrigation land (ha)	Water rights (%)	Monthly canal water discharge (m^3^/s)											
				January	February	March	April	May	June	July	August	September	October	November	December
1	Imam Sahib	7741.9	3.80	1.37	1.47	1.92	3.51	4.30	2.46	1.64	1.24	1.22	1.28	1.39	1.37
2	Nahar-i-Shahi	14,005.2	10.66	3.84	4.12	5.39	9.84	12.05	6.91	4.59	3.47	3.41	3.60	3.91	3.85
3	Sia Gird	7702.0	2.85	1.03	1.10	1.44	2.63	3.22	1.85	1.23	0.93	0.91	0.96	1.05	1.03
4	Balkh	8086.6	1.33	0.48	0.51	0.67	1.23	1.50	0.86	0.57	0.43	0.43	0.45	0.49	0.48
5	Moshtaq	9207.1	3.98	1.43	1.54	2.01	3.67	4.50	2.58	1.71	1.29	1.27	1.35	1.46	1.44
6	Chemtal	8003.4	3.12	1.12	1.21	1.58	2.88	3.53	2.02	1.34	1.01	1.00	1.05	1.14	1.13
7	Abdullah	22,866.3	13.32	4.80	5.15	6.73	12.29	15.06	8.63	5.74	4.33	4.26	4.50	4.88	4.82
8	Dawlat Abad	38,728.2	14.27	5.14	5.52	7.21	13.17	16.13	9.25	6.15	4.64	4.56	4.82	5.23	5.16
9	Char Bolak	36,078.3	14.27	5.14	5.52	7.21	13.17	16.13	9.25	6.15	4.64	4.56	4.82	5.23	5.16
10	Fayaz Abad	17,180.5	11.42	4.11	4.42	5.77	10.54	12.91	7.40	4.92	3.71	3.65	3.86	4.19	4.13
11	Aqcha	88,706.0	20.94	7.54	8.10	10.58	19.33	23.67	13.57	9.02	6.81	6.70	7.08	7.68	7.57
Total		258,305.5	100.00	36.00	38.66	50.51	92.26	113.00	64.78	43.06	32.50	31.97	33.77	36.65	36.14

A comparison between the allocated amounts of irrigation water in the newly developed scenario (Table 4) and those based on existing practices (Table 6) indicates that the current irrigation water distribution is unreliable and cannot meet the water requirements for crop irrigation. The farmers face water stress issues during March, April, July, and August, and surplus river water is available in the other eight months. The greatest water shortage occurs in March and April, when more than $369.9 \times 10^{6} \;{\text{m}}^{3}$ of water is needed for irrigation. The annual average flow in subbasin 10 was estimated as $1.578 \times 10^{9} \;{\text{m}}^{3}$ by the SWAT model from 2013 to 2018. Additionally, the irrigation water requirement in the BRB was estimated to be $1.197 \times 10^{9} \;{\text{m}}^{3}$. The Balkhab River transports enough water for the irrigation of existing agricultural lands and provides $381 \times 10^{6} \;{\text{m}}^{3}$ of surplus water each year. The water shortages come from the timing differences between the peak irrigation water demand and the peak river flow times.

**Figure 6:**
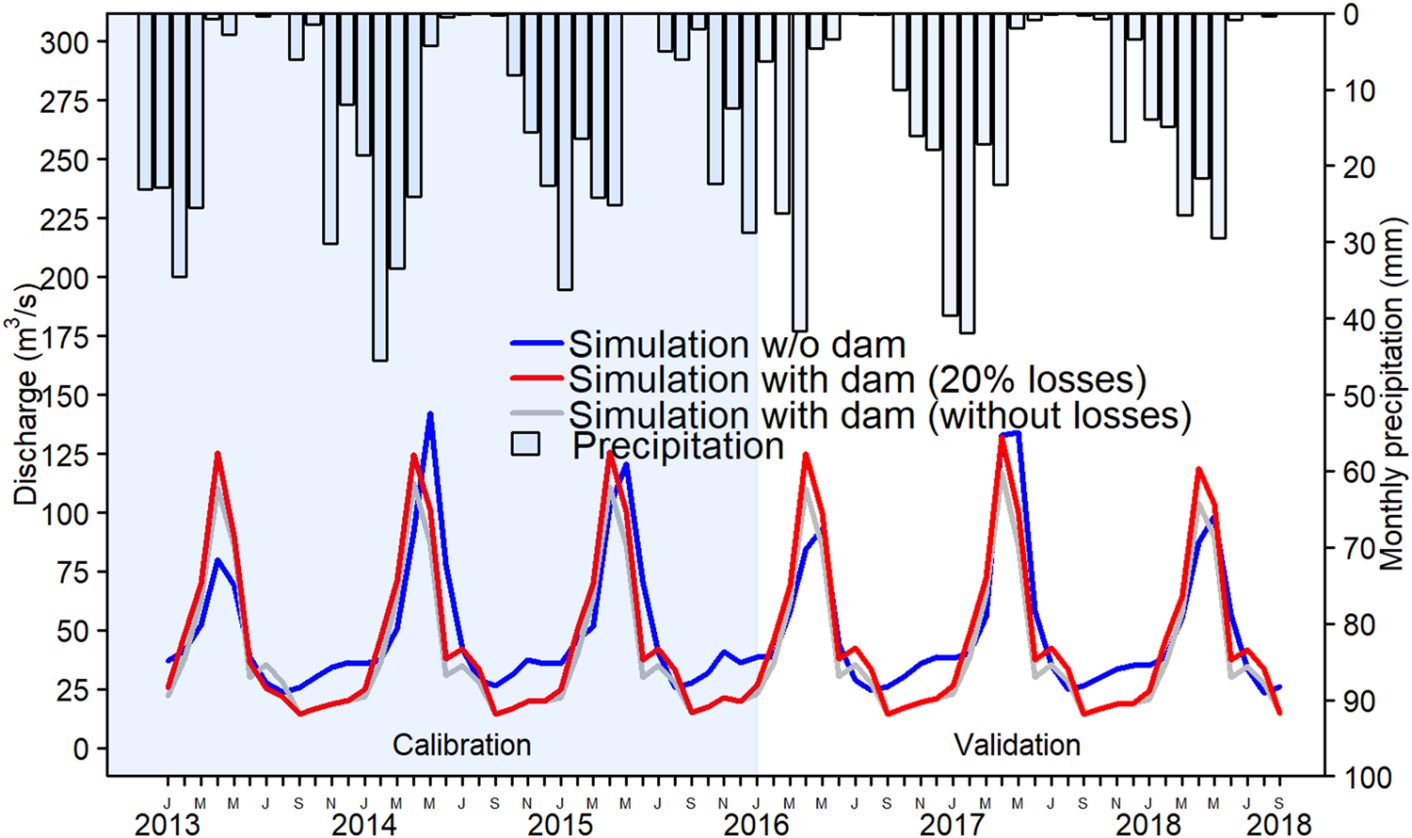
Resulting hydrographs from subbasin 7 without (blue) and with (red) dam inclusion in subbasin 10 with 20% irrigation water losses. The grey line indicates the result without the 20% losses in the modelling. (R version R- 4.1.2, drawn by author).

The proposed dam in subbasin 10 and the corresponding management scheme were added to the SWAT model. The hydrographs in Fig. 6 depict the river discharge in subbasin 7 before and after dam inclusion in the model. The resulting hydrographs reflect the water shortage during March and April from 2013 to 2018, and the basin experiences a small water shortage during July and August each year. As depicted in the hydrographs, May, when peak flows and floods occur, displays the highest potential for water storage. The Balkhab River has the potential to irrigate existing agricultural lands and promote further land development in the case of storing excess water during the eight months when irrigation water consumption is less than the available amount of water resources.

## Discussion

### Effects of the dam reservoir

Irrigation water consumption is highest during April in the BRB, and peak river discharge occurs in May. Additionally, river discharge cannot provide enough water in March, April, July, and August in the BRB. The Balkhab River transports enough water to irrigate the existing agricultural lands and promote the further development of agricultural land. During May, river discharge is increased by floods on certain days, and farmers cannot use these water resources. To overcome these challenges, a controlling hydraulic structure is needed. A dam control structure can store flood water for later consumption and balance the water supply and consumption demands during months with low river discharge.

In the case of dam construction in the BRB, there is potential to store water during the winter season, when agricultural water consumption reaches a minimum, and in May, when floods occur. The stored water can be managed and released based on the crop water requirements in each month to prevent the loss of agricultural products before harvesting. Such a scheme could not only increase the security of the agricultural sector and products but can also contribute to the economic development of the country and improve food security.

**Figure 7:**
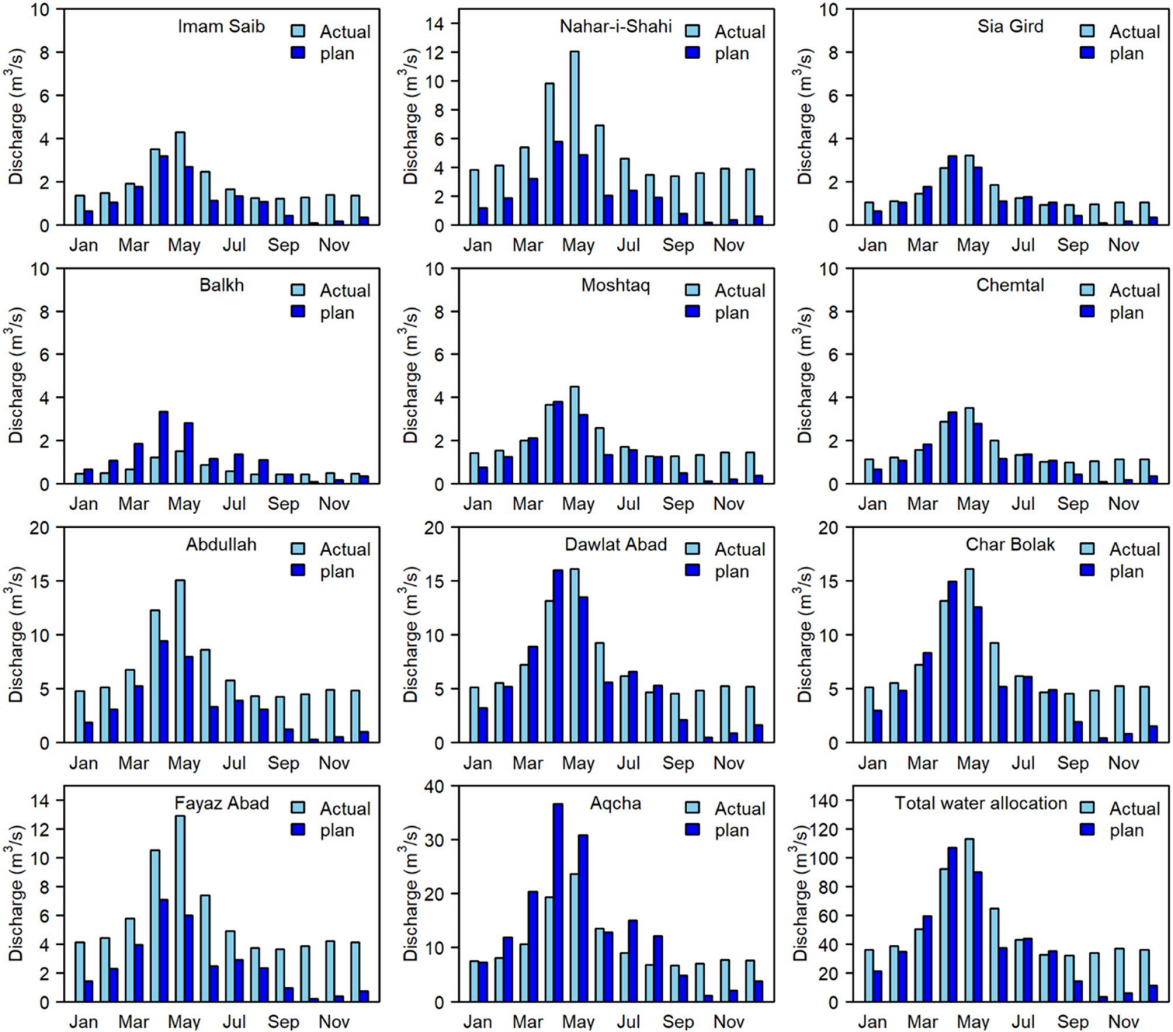
Comparisons of the current and planned water distributions based on water allocations in the 11 irrigation canals and the total water allocation. (R version R- 4.1.2, drawn by author).

Referring to the bar charts shown in Fig. 7, especially the final bar charts that show the total water allocation, it is clearly illustrated that the Balkhab River not only has the potential to irrigate the current arable land areas and newly developed agricultural lands. Based on the total water allocation amounts presented in Fig. 7, the discharge in the mainstream is higher than the amount of water required for consumption in the agricultural sector during January, February, May, June, September, October, November and December and lower than the required amount in other months.

### Agricultural water management

The existing traditional water management is effective since water users are engaged in determining the water distribution in irrigation canals. The *mirab* system is acceptable by the local community and government authorities. Water allocation through the main canals by the government and the current water allocation scheme based on tax payments (water rights) and agricultural land areas should be established with a more scientific approach than is currently used. The current water allocation approach results in poor irrigation downstream and an unequal distribution of water to farmers. Subbasin 1 encompasses 88,706 ha of arable land, and the total current arable land in the BRB is 296,744 ha. The water allocated to subbasin 1 through the Aqcha canal accounts for 20.4% of all allocated water, and the arable land in the subbasin accounts for 29.8% of all arable land in the basin. The Fayaz Abad canal transmits 11.42% of the allocated river water for 17,180.5 ha of arable land. This unequal water distribution has led to ecosystem degradation and the migration of farmers to large cities in subbasin 1.

Water resource management in the BRB must be reformed to ensure an equal water distribution among users. Such a scientific approach can promote stable and sustainable development in the agricultural sector. The unequal water distribution makes downstream farmers vulnerable and increases economic and food security risks. Moreover, unequal water allocation issues, drought, and extreme weather are the main challenges for farmers in rural areas in Afghanistan. The country cannot reach its energy, irrigation, or urban and rural development goals unless it makes substantial improvements in the development and management of water resources.

In Fig. 7, the current water distribution and that based on planned water management in the BRB after the proposed dam is constructed are compared; additionally, the uneven water distribution is visualized for water users. For example, the Imam-Saib canal receives more water than is required water for irrigation in its area, and the Aqcha canal receives less water than required for its area in February, March, April, May, July, and August. The uneven water distribution among water users has caused many farmers to migrate to large cities and neighbouring countries for work. Moreover, a large portion of river discharge comes from spring floods during April and May, and the agricultural sector does not require water during the winter season, when no irrigation is necessary. Thus, based on the current water management approach in the BRB, reform is needed to improve the irrigation scheme by adjusting water allocation amounts and storing water for later use.

### Agricultural land development

As mentioned in “Irrigation scenario with a proposed dam” section, the Balkhab River transports 381 million cubic metres of surplus water beyond what is needed for irrigating the existing agricultural lands. This extra water is wasted because the flows occur in the nonirrigation period in winter or during the short flood periods in the spring. Currently, 1.8 million ha of agricultural lands are cultivated in Afghanistan, and before 1980, approximately 3.3 million ha were cultivated^8^. Based on the data in Table 3, the water requirement of 1 ha of agricultural land in the BRB is $4559.5\;{\text{m}}^{3}$ annually. In the case of dam construction in the BRB, the potential exists to irrigate an additional 18,470.6 ha. In the case of dam construction, the area of arable land could increase by 6.18% in the BRB. The improvement associated with dam construction could highly affect farmers’ lives and agricultural productivity. Water losses in irrigation canals are another factor that must be considered in the future development of watersheds. The outputs in Fig. 6 show that in the ideal case of zero water losses in the irrigation canals the water resource can develop up to 83,561.8 ha of new agricultural lands in the study basin.

## Conclusions

In this study, the potential for irrigation in the BRB was determined to be adequate for the current agricultural lands, and an additional 18,470.6 ha of new agricultural land could also be irrigated. In the BRB, the traditional *mirab* system is applied for water distribution and canal maintenance. The assessments of the current policies for water management in the BRB indicate that the water distribution is unequal among farmers, and upstream agricultural lands use considerably more resources than downstream agricultural lands.

During March and April, wheat-cultivated lands, as the main cereal of the country, are at risk of water shortages in the BRB. Water scarcity issues can decrease the wheat yield or completely destroy the crop in these two months. Users cannot fully utilize the considerable potential of the water resources provided by the Balkhab River due to a lack of a control system during peak flows in the spring and winter flows; this water could be stored and used later for irrigation. The construction of controlling hydraulic structures and water resource storage during these seasons could mitigate water scarcity issues in normal years and drought periods.

The results of the current study can be utilized by policy makers to make decisions regarding water resource management in the study area. More accurate and individual studies are required for dam site selection, irrigation water allocation, and cost–benefit analysis prior to making final decisions related to dam construction. The current results can provide preliminary information for future short-term and long-term detailed studies in the BRB region in northern Afghanistan.

## Supplementary Information

Supplementary Table S1.

## Data Availability

Available upon requests.

## References

[1] United Nation. Water Scarcity. *UN Water*. https://www.unwater.org/water-facts/scarcity/ (2020).

[2] Seckler D, Barker R, Amarasinghe U (1999). Water scarcity in the twenty-first century. Int. J. Water Resour. Dev..

[3] Qutbudin, I. *et al.* Seasonal drought pattern changes due to climate variability: Case study in Afghanistan. *Water (Switzerland)* **11**, 1096 (2019).

[4] Zoljoodi M, Didevarasl A (2013). Evaluation of spatial-temporal variability of drought events in Iran using Palmer Drought Severity Index and its principal factors (through 1951–2005). Atmos. Clim. Sci..

[5] Ta, Z., Yu, R., Chen, X., Mu, G. & Guo, Y. Analysis of the spatio-temporal patterns of dry and wet conditions in Central Asia. *Atmosphere (Basel)* **9**, 7 (2018).

[6] Li Z, Chen Y, Fang G, Li Y (2017). Multivariate assessment and attribution of droughts in Central Asia. Sci. Rep..

[7] NSIA. *Afghanistan Statistical Yearbook*. 10.29171/azu_acku_musalsal_ha4570_6_alif2_seen28_v1363 (2019).

[8] Mahmoodi S (2008). Integrated water resources management for rural development and environmental protection in Afghanistan. J. Dev. Sustain. Agric..

[9] Rout, B. *Water Management, Livestock and the Opium Economy. How the Water Flows: A Typology of Irrigation Systems in Afghanistan* (2008).

[10] Viala, E. Irrigation management in Afghanistan: The tradition of Mirabs. In *Water Rights and Related Water Supply Issues, Salt Lake City, Utah, October 13–16, 2004* (Colorado State University Libraries, 2003).

[11] Ward FA, Amer SA, Ziaee F (2013). Water allocation rules in Afghanistan for improved food security. Food Secur..

[12] Salman D, Amer SA, Ward FA (2017). Protecting food security when facing uncertain climate: Opportunities for Afghan communities. J. Hydrol..

[13] Resilience, B. *The State of Food Security and Nutrition in the World*. *Rome: Building Resilience for Peace and Food Security* (2017).

[14] Dinar A, Tieu A, Huynh H (2019). Water scarcity impacts on global food production. Glob. Food Sec..

[15] Ullrich A, Volk M (2009). Application of the Soil and Water Assessment Tool (SWAT) to predict the impact of alternative management practices on water quality and quantity. Agric. Water Manag..

[16] Anand, J., Gosain, A. K. & Khosa, R. Optimisation of multipurpose reservoir operation by coupling soil and water assessment tool (SWAT) and genetic algorithm for optimal operating policy (case study: Ganga River Basin). *Sustainability* **10, **1660 (2018).

[17] Panagopoulos Y (2014). Assessing the cost-effectiveness of irrigation water management practices in water stressed agricultural catchments: The case of Pinios. Agric. Water Manag..

[18] Dechmi F, Burguete J, Skhiri A (2012). SWAT application in intensive irrigation systems: Model modification, calibration and validation. J. Hydrol..

[19] Santhi C, Srinivasan R, Arnold JG, Williams JR (2006). A modeling approach to evaluate the impacts of water quality management plans implemented in a watershed in Texas. Environ. Model. Softw..

[20] Logan, T. A. *et al.* Radiometrically terrain corrected ALOS PALSAR Data available from the Alaska Satellite Facility. *AGUFM* **2014**, IN33B-3762 (2014).

[21] FAO. *The Islamic Republic of Afghanistan Land Cover Atlas* (UNFAO, 2016).

[22] Abbaspour KC, Vaghefi SA, Yang H, Srinivasan R (2019). Global soil, landuse, evapotranspiration, historical and future weather databases for SWAT applications. Sci. Data.

[23] Batjes NH (1997). A world dataset of derived soil properties by FAO–UNESCO soil unit for global modelling. Soil Use Manag..

[24] Doe J (1962). Soil map of the world. Soil Horiz..

[25] NRB. *[Water Management of the Northern River Basins]* (2019).

[26] FAO. *Cropping Calendar—Dates of Planting and Harvesting, Northern Afghanistan (Faryab, Jawzjan, Saripol, Balkh, Samangan).* http://www.fao.org/tempref/GI/Reserved/FTP_FAAFG/pubs_cal2.htm (FAO, 2009).

[27] Murthy CS, Raju PV, Jonna S, Hakeem KA, Thiruvengadachari S (1998). Satellite derived crop calendar for canal operation schedule in Bhadra project command area, India. Int. J. Remote Sens..

[28] Wali E, Tasumi M, Shinohara Y, Takeshita S (2019). Mapping crop types and the crop water requirements over small-sized irrigated fields in the Khost Province of Afghanistan. J. Rainwater Catchment Syst..

[29] Clemente, P., De Luca, D. A., Dino, G. A. & Lasagna, M. Water losses from irrigation canals evaluation: Comparison among different methodologies. In *EGU General Assembly Conference Abstracts*, vol. 15, 2013–9024 (2013).

[30] Anderson, I. M. *Rehabilitation of Informal Irrigation Systems in Afghanistan*. Design Manual, 1st edn, 68–95 (United Nations Food Agric. Organ. (FAO), 1993).

[31] Rout, B. *How the Water Flows: A Typology of Irrigation Systems in Afghanistan* (Citeseer, 2008).

[32] Duan, Y. *et al.* Inclusion of modified snow melting and flood processes in the SWAT model. *Water (Switzerland)* **10,** 1715 (2018).

[33] Grusson Y (2015). Assessing the capability of the SWAT model to simulate snow, snow melt and streamflow dynamics over an alpine watershed. J. Hydrol..

[34] Arnold, J. G., Srinivasan, R., Muttiah, R. S. & Williams, J. R. Large area hydrologic modeling and assessment part I : Model development’ basin scale model called SWAT (Soil and water speed and storage, advanced software debugging policy to meet the needs, and the management to the tank model) Sugawara et al., 1. **34**, 73–89 (1998).

[35] Guiamel IA, Lee HS (2020). Watershed modelling of the Mindanao River Basin in the Philippines using the SWAT for water resource management. Civ. Eng. J..

[36] Adu JT, Kumarasamy MV (2018). Assessing non-point source pollution models: A review. Polish J. Environ. Stud..

[37] Reddy, V. R. Watershed management in Afghanistan : Lessons from South Asia. In *From Catchment Management to Managing River Basins*, vol. 1, 55–85 (Elsevier Inc., 2019).

[38] Guiamel IA, Lee HS (2020). Potential hydropower estimation for the Mindanao River Basin in the Philippines based on watershed modelling using the soil and water assessment tool. Energy Rep..

[39] Abbaspour, K. C. User manual for SWAT-CUP, SWAT calibration and uncertainty analysis programs. *Swiss Fed. Inst. Aquat. Sci. Technol. Eawag, Duebendorf Switz.* **93** (2007).

[40] Homan JW, Luce CH, McNamara JP, Glenn NF (2011). Improvement of distributed snowmelt energy balance modeling with MODIS-based NDSI-derived fractional snow-covered area data. Hydrol. Process..

[41] Hussainzada W, Lee HS (2021). Hydrological modelling for water resource management in a semi-arid mountainous region using the soil and water assessment tool: A case study in northern Afghanistan. Hydrology.

[42] Nash JE, Sutcliffe JV (1970). River flow forecasting through conceptual models part I—A discussion of principles. J. Hydrol..

[43] Gupta HV, Sorooshian S, Yapo PO (1999). Status of automatic calibration for hydrologic models: Comparison with multilevel expert calibration. J. Hydrol. Eng..

[44] Moriasi DN, Gitau MW, Pai N, Daggupati P (2015). Hydrologic and water quality models: Performance measures and evaluation criteria. Trans. ASABE.

[45] Hussainzada Wahidullah, Lee Han Soo, Vinayak Bhanage, Khpalwak Ghulam Farooq (2021). Sensitivity of snowmelt runoff modelling to the level of cloud coverage for snow cover extent from daily MODIS product collection 6. Journal of Hydrology: Regional Studies.

