# Supplementary material for: Effect of an improved agricultural irrigation scheme with a hydraulic structure for crop cultivation in arid northern Afghanistan using the Soil and Water Assessment Tool (SWAT)
Source: Sci Rep. 2022 Mar 25;12:5186. doi: 10.1038/s41598-022-09318-2 (PMC8956590; doi:10.1038/s41598-022-09318-2)
Supplement: Supplementary file 1 — Supplementary Table S1. [file 41598_2022_9318_MOESM1_ESM.docx]

**Supplementary information**

**Table S1.** Monthly and seasonal crop water requirements (mm) by crop type (Wali et al., 2019)

| **Crop type** | **Jan** | **Feb** | **Mar** | **Apr** | **May** | **Jun** | **Jul** | **Aug** | **Sep** | **Oct** | **Nov** | **Dec** | **Seasonal** |
| --- | --- | --- | --- | --- | --- | --- | --- | --- | --- | --- | --- | --- | --- |
| Wheat | 32.9 | 52.2 | 79.1 | 127.5 | 93.9 |  |  |  |  |  | 9.0 | 17.1 | 411.7 |
| Clover | 39.5 | 43.9 | 61.2 | 57.6 |  |  |  |  |  |  | 32.0 | 35.7 | 269.9 |
| Vegetables (spring) |  | 16.8 | 50.1 | 116.4 | 93.6 |  |  |  |  |  |  |  | 276.9 |
| Vegetables (summer) |  |  |  |  |  | 87.7 | 122.5 | 135.4 | 105.1 | 5.8 |  |  | 456.5 |
| Maize |  |  |  |  |  | 22.0 | 84.8 | 149.9 | 120.3 | 37.5 |  |  | 414.5 |
| Mung bean |  |  |  |  |  | 16.1 | 98.8 | 148.2 | 66.8 |  |  |  | 329.9 |
| Rice |  |  |  |  | 55.7 | 153.9 | 171.0 | 154.8 | 123.7 | 62.5 |  |  | 721.6 |
| Cauliflower |  |  |  |  |  | 51.4 | 104.7 | 113.4 | 108.4 | 89.3 | 12.2 |  | 479.4 |
| Orchards |  | 14.6 | 47.5 | 109.0 | 136.3 | 138.3 | 139.4 | 122.6 | 47.9 |  |  |  | 755.6 |
| Peanut |  |  |  |  |  | 59.0 | 113.1 | 147.5 | 104.9 | 10.6 |  |  | 456.3 |
| Soybean |  |  |  |  |  | 88.2 | 167.3 | 148.4 | 116.2 | 42.5 |  |  | 583.8 |
